# The handling of evidence in national and local policy making: a case study of alcohol industry actor strategies regarding data on on-premise trading hours and violence in Norway

**DOI:** 10.1186/s12889-018-6348-y

**Published:** 2019-01-09

**Authors:** Ingeborg Rossow, Jim McCambridge

**Affiliations:** 1 Department of Alcohol, Drug and Tobacco Research, Norwegian Institute of Public Health, POB 4404 Nydalen, N-0403 Oslo, Norway; 2 Department of Health Sciences, University of York, York, UK

**Keywords:** Alcohol industry, Alcohol policy, Evidence, Content analysis

## Abstract

**Background:**

Effective alcohol policy measures conflict with the interests of the alcohol industry. In this study we addressed how various alcohol industry actors in Norway have responded to research findings and police data relating to the possible impacts of changes in on-premise trading hours on violent offending.

**Methods:**

A content analysis of documents was undertaken. The documents comprised i) hearing statements from policy processes on on-premise trading hours at the national level, and in 15 Norwegian cities, and ii) newspaper articles and other media coverage of this topic in Norway.

**Results:**

Alcohol industry actors employed a range of strategies to shape the use of evidence regarding on-premise trading hours and violence. Nationally, the relevance of the international research literature was questioned before the publication of an unfavourable national study which was criticized directly. This led to commissioned attacks on the findings, constructing what were claimed to be disagreements between experts, emphasis on the complexity of violence and the role of confounding variables, and deflecting attention to alternative interventions. The handling of evidence at the local level was importantly different, where different industry actors and forms of evidence, notably police data, were involved in debates.

**Conclusion:**

Alcohol industry actors employed various strategies to shape perceptions and use of evidence to advance their interests. The particular strategies and arguments changed over time as new data and research became available, and also varied between the national and the local levels, and by categories of industry actors.

## Background

Alcohol use is among the leading causes of disease burden globally [1]. Alcohol control policies can regulate the economic and physical availability of alcohol, impact on alcohol sales, alcohol consumption and alcohol related harm [2, 3]. Thus, using effective alcohol policy measures to curb sales and consumption may on the one hand reduce alcohol related health and social problems, and on the other hand, compromise the economic interests involved in the production and sale of alcoholic beverages. This illustrates an inherent conflict of interests between public health and the alcohol industry. Similar conflicts of interests are found in many other areas, including tobacco, gambling, food, and environmental pollution [4–6].

Examining both alcohol producers and other industries, Jahiel and Babor [4] identify common patterns in how industrial corporations respond to perceived threats to commercial activities linked to health problems: ranging from silence about a health problem linked to a product to the commissioning of industry-funded research to cast doubt on scientific findings. The literature on tobacco industry activities regarding evidence shaping has identified a number of strategies including: funding research and paying scientists as advisors or spokespersons, cherry picking of data that favours the industry, criticizing evidence and emphasizing its complexity and uncertainty, and emphasizing disagreement among scientists and focusing on doubt in science [7, 8]. The latter is well illustrated by Oreskes and Conway [9] and Michaels [10], who showed how the tobacco companies have used scientists to instil doubt about research findings linking tobacco use to lung cancer and other health harms. Subsequently, the energy industry is known to have extensively funded research casting doubt about human actions that have caused global warming in a similar manner, and involving some of the same key individuals [9]. McGarity and Wagner [11] identified six types of strategies employed by advocates of various industries, including the tobacco, food and energy industries, to ‘bend science’ to protect their economic interests at the expense of public health interests. These strategies are similar to, though arguably offer a more comprehensive framework than the abovementioned strategies, and they are briefly summarized in Table 1.

**Table 1:** Strategies for bending science: categories and examples

Overview of strategies for bending science: Categories and examples^a^	
Shaping science:	Creating research to fit one’s needs; e.g. manipulating study design, research data and methods
Hiding science:	Concealing unwelcome information, e.g. pharmaceutical industry hiding results from own research, demonstrating adverse effects of their products
Attacking science:	These strategies are often in terms of ‘post-publication damage control’, particularly targeting policy-makers and the public, attacking study methods creating doubt about study validity: a) Turning reliable research into ‘junk’; e.g. claim research as ‘fatally flawed’ based on limited scientific grounds and voiced by hired experts b) illegitimate obfuscatory attacks; e.g. raising hypothetical charges about research design that are not supportable and not easily refuted; c) unbalanced attacks; e.g. allied attack where third parties without industry connection (think tanks) are engaged on the industry friendly side.
Harrassing scientists	a) Challenge integrity of researchers, e.g. as publicized attacks b) Draining resources through lawsuits or unreasonable and burdensome demands for data and documents
Packaging science	Assembling expert group to advance favoured outcome, e.g. by commissioning publications summarizing the state of science, which ignores or belittles unwelcome research.
Spinning science	Manipulating public perceptions about credible science, e.g. campaigns to generate pressure on decision-makers to discount it.

^a^Based on McGarity and Wagner, 2008: Bending science. How special interests corrupt public health research

Whether the alcohol industry has played a similar role with respect to research on effective alcohol policy measures is less well studied. Bakke and Endal [12] found in four sub-Saharan African countries that the alcohol industry had produced early drafts of national alcohol policy documents that ignored the international scientific literature on the effectiveness of price and availability measures. Babor [13] reported three examples where scientists had been paid, or offered payment, by the industry for attacking research on alcohol control policies. Petticrew and colleagues [14] and McCambridge et al. [15], identified the conduct of weak evaluation studies as a basis for exaggerated claims about the effectiveness of community alcohol partnerships involving industry actors. The latter study also identified serious misrepresentations of the scientific literature in attempts to influence national policy in Scotland. A recent systematic review of alcohol industry involvement in policy making identified that industry actors “fund or disseminate policy relevant research with supportive findings to create a separate, circumscribed and self-referential literature using think-tanks, academics, consultancies and similar policy actors” in order to influence policy [16]. A related systematic review on alcohol industry involvement in science drew attention to legitimation and public relations benefits [17]. This review identified instrumental management of research for the purposes of policy influence by industry actors, and longstanding and unresolved concerns about the activities of organisations funded by the global alcohol producers in particular. How alcohol industry actors respond to research on alcohol policy measures whose findings are in conflict with industry interests is thus one strand of an emerging literature on the use of evidence in alcohol policy making.

In Norway, local authorities (at the municipality level) decide on licenses for off-premise and on-premise alcohol sales and thus on outlet density and trading hours, within nationally determined parameters. Every 4 years, each city/municipality council is expected to review the local alcohol action plan, which includes regulations on alcohol outlet density and trading hours. This permits ‘the policy window’ for local alcohol policy changes to open regularly [18, 19].The issue of on-premise closing hours is prominent in media attention, policy hearing statements and other involvements of various stakeholders in the policy making processes [18]. The nationally determined parameters for trading hours are stated in the Alcohol Act, and changes to this act can be made as a result of a process including a reasoned proposal and review of hearing statements from various parties that may be affected. Such a process occurred in 2009–2012. We therefore investigated on-premise closing hours as an illustrative case study of the dynamics of evidence use in alcohol policy – both at the national and at the local level – with particular attention to industry actors in the present study.

A key feature of the public debates on on-premise trading hours in Norway is the possible impacts of trading hours on violence. In September 2009, the Ministry of Health and Care proposed, among a number of changes to the Alcohol Act, that the national on-premise latest permitted trading hours (hereafter “closing times”) were to be reduced by one hour, from 3.00 am to 2.00 am. This reflected evidence in the international research literature that earlier closing times led to less violence [20–22], though most such evidence pertained to the effects of implementing larger changes (e.g. of 2 h or more) [21, 22]. The Ministry proposal stated that: “A review of international research shows that changes in on-premise trading hours are accompanied by changes in violence rates. When trading hours increase, so do violence rates, and the other way around.” [23]. The proposal was sent for consultation and a year later, in December 2010, the Ministry concluded on the basis of hearing statements from an exceptionally large number and broad range of actors, that they would await to make a decision until an ongoing Norwegian research project examining the impact of trading hours on violence was finished [24].

The abovementioned research project was initiated and led by the present first author and examined the impact of restricting or extending closing times between half an hour and one and a half hours on violence rates in 18 Norwegian cities (hereafter the RN study). Quarterly data on police reported violent assaults at nighttime on weekends in city centers over a 10 year period in these cities were analyzed [25]. Assaults outside the city center during the same time window were used as proxy for potential confounders. The findings were robustly established across three different statistical modelling techniques. In addition to the peer review process of a leading specialist journal, three independent post-publication assessments of the study were obtained [26]. A subsequent systematic review stated: “The most comprehensive study of late-night trading hour changes comes from Norway, where Rossow and Norström examined the impact of small changes (< 2 h) in allowable late-night trading for bars in 18 Norwegian cities. They found that each 1-h change in trading hours was associated with a change of 16% in recorded assaults. This is the only study to include both extensions and restrictions on trading hours, and the findings were similar for changes in both directions, adding more evidence that effects were causally related to the policy changes” [27].

The publication of this study in September 2011 was immediately relevant to both the on-going decision-making on national latest permitted trading hours and for decisions at local levels in the next few months, as most municipalities were about to revise their alcohol action plans and thereby decide on possible changes in local closing times. In addition to the formal research, the local police’s own violence statistics was another key data source for assessment of the possible impacts of changes in on-premise closing times that was prominent in media discussions of these issues.

## Methods

In this study, we examine how various alcohol industry actors at both the national and local level in Norway responded to these two contrasting forms of evidence; the research findings and the police statistics relating to the possible impacts of changes in on-premise trading hours on violent offending.

Three data sources were used as follows; i) policy documents including formal hearings statements from policy making processes at the national and the local level; ii) Norwegian print and ether media articles; and iii) sources further identified from media articles.

Documents from the national policy making process were obtained from the Norwegian government’s website (regjeringen.no), while those on local policy making processes were obtained from the municipality web-sites for 19 of the 30 largest cities in Norway. In all 19 cities, on-premise trading hours were on the political agenda in the local alcohol policy making processes in 2011–2012. Virtually all Norwegian newspapers (national, regional and local) (*n* = 107) and the main radio and TV channels (*n* = 7) were electronically searched in a media database (Retriever ©) using the following search terms; ‘On-premise trading hours*’ AND ‘Violence*’ AND various search terms for actors in the alcohol industry AND ‘Police*’, or ‘statistics*’, or ‘research*’, covering the period 01.01.2009 through 01.09.2012. This period was selected because the debates surrounding the decision making process over a possible national restriction on maximum trading hours started early in 2009 and the final decision was announced in June 2012. At the local level, on-premise trading hours were likely to be politically debated and possibly changed in the wake of newly elected municipality councils in most Norwegian municipalities (elections held in September 2011), i.e. within the data collection period.

Alcohol industry actors included individual owners and managers of on-premise licenses (e.g. pubs, nightclubs), the national and regional trade organizations in the hospitality industry, individual alcoholic beverage producers (e.g. breweries) and their national trade organizations, and the trade organization of wholesalers/importers. Other types of commercial actors that may be affected by on-premise trading hours changes (e.g. taxi services and travel agencies/event agencies) were not included.

Only material of relevance to how alcohol industry actors dealt with data on the closing times – violence association was included. In the media articles, data on responses to scientific and non-scientific evidence (police data) comprised both direct and indirect quotations from alcohol industry actors and journalistic interpretation of these responses when this was supported in other parts of the article.

The media search strategy identified a total of 2825 media articles, newspaper contributions/letters to the editor, and other media pieces. Headings and extracts of these were all screened and approximately half were read in full text. Altogether 56 unique media articles (including newspaper contributions) related to industry responses to evidence on closing times and violence. Similar versions to those included were identified in other media sources, and are not counted within the 56 selected for study. In addition, numerous media articles covered industry representatives’ views on on-premise trading hours as an issue, but did not express views on research evidence or police statistics specifically and were thus excluded.

The media data were sorted by date and categorized by i) policy level (national vs local); ii) type of industry actor (national/regional organization vs local individual); and iii) type of evidence (research vs police statistics); and iv) media reach (national vs local). Initially, we applied a summative content analysis [28] to explore whether the two main categories of industry actors (organizations vs local individual actors) differed with respect to involvement at the two policy levels, type of evidence in focus, and appearance in local or national media. Next, we applied a directed content analysis, guided by previous empirical studies of industry strategies to shaping evidence, employing a classification process of coding and identification of patterns in the text data [28]. The first author approached the data chronologically and separately for the national and the local policy level. The first author explored whether there was consistency or dominance in the data both across industry actors and over time, and whether there was heterogeneity in these respects. The second author discussed the analyses conducted by the first author at various points in the process. The main data source for these analyses was the media articles and hearing statements, but the industry commissioned reports provided some additional information.

## Results

**Fig. 1:**
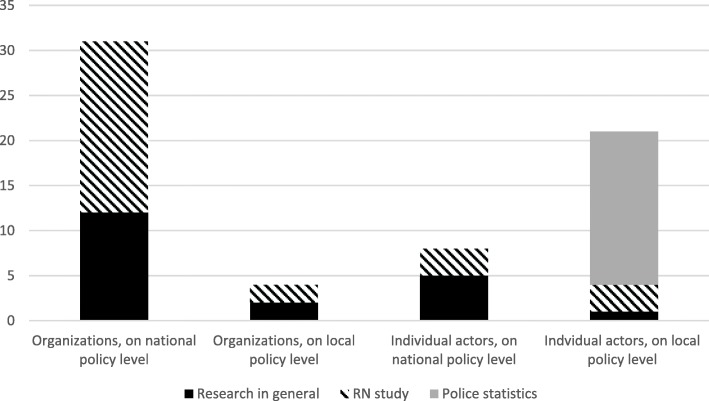
Count of media articles by industry actor types and levels of policy making

Different actors had different foci. In the media articles, there was primarily attention to research evidence at the national rather than the local level. The main industry organizations were more involved with research evidence and at the national level, whereas individual industry actors were more often concerned with the evidence at the local level, where police statistics were the predominant source of evidence discussed (Fig. 1). Moreover, the industry organizations mainly expressed their views in national news media with large circulation and often as newspaper contributions, whereas individual actors, reflecting on evidence pertaining to local policy, typically expressed their views in the local media. There was no evidence of any activities by the global producers or their organizations.

Hearing statements from industry actors regarding possible national restrictions (*n* = 10), included eight statements referring to the evidence on trading hours and violence. At the local level, industry actors gave hearings statements in 9 of the 19 cities where on-premise trading hours were on the political agenda, and among these 9 hearing statements, a national hospitality industry trade organization gave 2 statements addressing evidence on closing times and violence. In the hearing statements, the trade organizations were typically involved at the national level paying attention to research evidence, whereas at the local level, there were few hearing statements from industry actors, concerned with research evidence (*n* = 1) and police statistics (*n* = 2) respectively.

The two main national hospitality industry trade organizations, NHO Reiseliv and Virke (formerly HSH), each commissioned reports on violence in relation to on-premise trading during the autumn 2011/winter 2012. These reports were both concerned with the possible national restrictions.

### International research evidence use in responses to the proposed national restrictions on closing times

Various industry actors provided hearing statements, representing the hospitality industry trade organizations, the breweries’ organization, the wine and liquor wholesalers and importers’ organization, the groceries’ wholesalers’ organization, and a few individual actors in the hospitality industry, and all argued strongly against the proposal. The policy arguments included: expected economic losses for the hospitality industry; the municipalities’ right to decide on local policy; and that other strategies would be better suited to curb violence and nuisance. A prominent argument was the lack of sufficient evidence, particularly in the Norwegian context. In eight of 10 statements, the evidence, as presented by the Ministry in their proposal, was criticized and refuted. Several statements were quite lengthy and paid substantial attention to this issue, particularly the statements from the larger organizations. Some actors simply stated that there was no evidence, whereas others conveyed a more detailed critique, and it was argued that evidence from the international literature was of little or no relevance in the Norwegian context, as the empirical studies pertained to larger changes in trading hours and/or came from other countries.

*“There is not a single research report in the entire world concluding that a small restriction in trading hours will lead to less violence” (Pub consortium, Bergen).*

*“The international research [ ] is in no way relevant in the debate of a one hour restriction.” (Utelivsbransjen, an organization of dance and music venues)*

At this time, these claims were, in one sense, largely in line with international reviews of the literature, which mainly showed harmful effects of large extensions of trading hours and few studies with inconsistent findings examining smaller changes in trading hours [20–22].

The proportion of total alcohol volume being consumed on-premise was emphasized in several statements, the argument being that any changes in this small volume was unlikely to impact on violence rates. Moreover, the complexity of the problem at hand was also noted by one actor, who claimed this meant a different approach was needed.

*“Harms from alcohol are a far more complex issue than the result of extended trading hours to 3.00 am. Complex issues demand complex solutions” (HSH,* a national hospitality industry trade organization*).*

There are some examples that industry actors had searched for alternative expert opinion so as to cherry pick statements from researchers or research publications in support of their view.

*“Violence researcher [named] does not believe trading hours and universal prevention measures are of any importance.” (Pub consortium owner, Bergen).*

Industry actors chose carefully which evidence to contest; although industry actors denied the existence of research evidence of an impact of trading hours on violence, the evidence of an overall association between alcohol consumption and violence was acknowledged. Industry actors were often rhetorically explicit in welcoming further research on this issue, thus not challenging the potential value of research evidence per se. Moreover, in several hearings statements, particularly those from the larger organizations, the actors portrayed themselves as serious and responsible, sharing the concern with the problem of violence in the night time economy, and being eager to find solutions.

In November 2010, when the Ministry of Health and Care announced their decision to delay national restrictions until they had Norwegian research evidence, industry actors expressed unanimous appreciation of the decision and repeated their views on the international evidence.

*“We have persistently noted the lack of sufficient evidence on the association between trading hours and violence. […] The Government deserves praise for having reached a responsible decision.” (NHO Reiseliv, hospitality industry trade organization*, *November 25, 2010).*

### National research evidence use in responses to the proposed national restrictions on closing times

Less than 1 year later, the RN study [25] was published, and first reached public attention in Norway on September 29, 2011 with front page coverage in a national newspaper. In the following weeks, NHO Reiseliv was prominent in the responses by industry actors. Their immediate response, on the same day, was that the issue was more complex than suggested and that the study had “*omitted taking other factors, including police on the streets and public transportation, into consideration*”.

Three days later, NHO Reiseliv nuanced and strengthened their critique by referring to a short manuscript, which they had commissioned from a private consultancy firm (Menon Business Economics). The manuscript, assessing the RN study, was leaked to the media and its main points conveyed via media interviews with NHO Reiseliv. The headline of the 1.5 page manuscript was “Large and important weaknesses in the [RN study] report”, a phrase that was repeatedly echoed by NHO Reiseliv and other hospitality industry representatives in the media. The main critique pertained to the methods employed in the RN study and claimed lack of control for other factors impacting on violence. The manuscript was later included as an appendix in a report from Menon Business Economics, also commissioned by NHO Reiseliv, which reviewed literature and Norwegian data related to on-premise trading hours, alcohol consumption and violence including the RN study [29]. NHO Reiseliv emphasized that the critique of the RN study, as if this had been convincingly demonstrated, meant there was an absence of evidence to support restricted trading hours.

*“Now that large and important weaknesses of the [RN] study have been revealed, it is impossible for policy makers to adopt restricted trading hours.” (NHO Reiseliv, October 1, 2011).*

The industry commissioned critique of the RN study gained support from a local level politician, who offered similar critical remarks in newspaper interviews. He elaborated these remarks and wrote a blog post on a right wing liberal think tank website [30] . Statements from the blog post attacking the science and devaluing its value for policy, like *“Useless report about on-premise trading hours”* and “*the findings are useless for politicians”* were frequently repeated by hospitality industry actors. Holding a PhD in meta-analysis of British government support, this politician was regarded by several journalists, and claimed by industry actors, to be an expert. Some commentators noted that the politician likely had an underlying political agenda, as he and his party were strongly in favour of extended trading hours. Nevertheless his critique fueled the media’s attention to what was now being construed as a disagreement between experts, and resulted in newspaper headlines like “*Strong fight between experts” (Dagbladet (a national newspaper), October 6, 2011).* This framing was also conveyed by industry actors in media interviews. Referring to the local politician’s blog post and to the authors of the commissioned review of the RN study, NHO Reiseliv emphasised what was presented as a convergence of expert opinion as follows.

*“Two other experts, independent of each other, have now found that there are large and important weaknesses of the [RN] study.” (NHO Reiseliv, October 6, 2011).*

The authors of the RN study (including the present first author) noted publicly that factors other than trading hours may also impact on violence rates, whilst defending the methods and study findings [31]. In a newspaper interview, NHO Reiseliv responded by stating: *“This confirms what we’ve been saying all the time, the issue is more complex than what the researchers claim. Now it seems that the researchers admit we were right in our critique”* (October 22, 2011).

Beyond attacking the science, another kind of critique pertained to research integrity and possible political manipulation of the research process. NHO Reiseliv claimed that the Minister of Health, being in charge of the research institute where the RN study was conducted, had been *“lucky to receive a conclusion she wanted”* (as described in a national newspaper editorial, September 30, 2011). In several debates on TV, this trade organization referred to the RN study in ways suggesting that the researchers served a political agenda. Thus not only was the integrity of the scientists attacked, the national politicians involved were also attacked.

The issue of credibility of the science and the scientists was linked to that of complexity, which was repeatedly brought up by NHO Reiseliv and other hospitality industry representatives, suggesting that it was unlikely that there could exist policy measures with the effects observed in the research.

*“We do not believe the world is so simple that violence is reduced by almost 20% if closing hours are restricted by one hour. A number of other factors impact on violence.” (NHO Reiseliv, October 20, 2011).*

In February 2012, the other large national hospitality industry trade organization (Virke, formerly called HSH) and a regional trade union for servers published a report, which is likely commissioned work from a consultancy firm [32]. This report reviewed briefly the international literature and it repeated the previously published critique by the local politician on the RN study, paying particular attention to alleged methodological weaknesses. The report concluded that the scientific evidence for impact of small changes in on-premise trading hours was weak and inconclusive and a number of other strategies to combat violence related to on-premise drinking were suggested.

*“Both Norwegian and international research demonstrate large uncertainty regarding the association between on-premise trading hours and violence, and especially when the regulation is less than two hours.” (Joint report from Virke and Oslo Server Union, February, 2012).*

The launch of this report received much media attention and further strengthened the voices calling for approaches other than earlier closing times, including having police dogs at bar entrances and offering free coffee when alcohol serving was finished. Such measures have no foundation in any evidence-base. Thus, over a 5 month period after the RN-study was published, the hospitality industry had commissioned two reports on on-premise trading hours and violence including a specific critique of the RN study and given numerous media interviews on this issue (*n* = 24). The arguments were no longer that evidence was lacking, but that the Norwegian research showing an impact of closing hours was methodologically flawed and that the researchers had a political agenda. Furthermore, it was asserted that Norwegian experts in the field disagreed, and that the problem of violence was much more complex than suggested by an implausibly simple solution of restricted closing hours (see Table 2). Unevidenced alternatives were promoted instead.

**Table 2:** Overview of handling of evidence by the industry observed in the present study

Strategies for bending science^a^	Any evidence in present study?
Shaping science	No
Hiding science	No
Attacking science:	Yes
a) Claim research as ‘fatally flawed’ based on limited scientific grounds and voiced by hired experts b) Illegitimate obfuscatory attacks c) Allied attack where third parties without industry connection (think tanks) are engaged on the industry friendly side.	a) Industry commissioned reports characterized the RN-study as having ‘large and important weaknesses’ b) Cherry picking data, selecting anecdotal evidence supporting the industry’s views c) RN-study criticized by liberal politician in think-tank website
Harrassing scientists a) Challenge integrity of researchers, e.g. as publicized attacks	Yes (not B, see Table 1) a) RN-study researchers accused of lip-serving the Minister of Health. (The police were also accused of manipulating routine data).
Packaging science	Yes
Commissioning publications summarizing the state of science, which ignores or belittles unwelcome research.	The hospitality industry commissioned two reports; both ignoring or belittling unfavourable evidence.
Spinning science	Yes
Manipulating public perceptions about credible science, framing the issue	Systematic framing in media: - of research evidence as flawed and therefore to be discounted in the policy-making; − constructing disagreement between “experts”; − emphasizing the complexity of violence and alternative ways to curb violence

^a^Based on McGarity and Wagner, 2008: Bending science. How special interests corrupt public health research

Up to early June 2012, just before the Norwegian Government was expected to finally decide whether or not to restrict the national latest permitted closing time from 3.00 am to 2.00 am, the wine and spirits importers’ association and NHO Reiseliv were involved in lobbying, as reported in the media. In one national newspaper article, representatives of both these organizations confirmed lobbying or “spreading information in society” by means of meetings with politicians, media reports, websites and multiple newspaper contributions and media interviews. Our data comprise only the latter two sources.

“*There is no factual evidence for the claim that closing earlier will lead to fewer violence incidents. However, there is reason to believe that many different efforts will lead to a reduction in violence and nuisance” (the Norwegian wine and spirits importers’ association, newspaper contribution to 5 national newspapers, June 2012)*

In late June 2012, the Norwegian government launched a white paper on alcohol and drug policy, which stated that the national maximum trading hours (3.00 am) were to be kept as they were. The Minister of Health made little comment on this, beyond stating that over-serving was the big problem and that the requirements for local control of bars and pubs were to be stricter.

### Evidence use in local policy making processes

In many Norwegian cities, the question of whether or not to restrict local closing times was being debated, alongside the national decision making process. Compared to the debate at the national level, as described above, a broader range of actors were involved in the local debates and local policy processes. These included individual bar owners/managers, local trade organizations and a local brewery, in addition to the national and regional trade organizations.

In the local debates, bar and pub owners rejected research on trading hours and violence, but mainly by stating mere disbelief in the findings. However, a local brewery referred to one of the industry commissioned literature reviews [32] in their hearing statement and stated that “g*iven the lack of scientific evidence for the effects of the proposed restriction, we ask that the current trading hours are continued*.” In a hearing statement requesting extended trading hours in one city, the regional hospitality industry trade organization (NHO/NiT) used a similar argument: “*there is no valid evidence that restricted trading hours has led to less violence*”. They further argued that use of both available research and the police statistics had not taken into account other factors that may have impacted on violence rates. A similar argument was used by a local brewery in their hearing statement responding to a proposed restriction of closing time.

This focus on police statistics on violence was much more prominent at the local than at the national level (see Fig. 1). In many cities, the police referred to their own statistics when they argued strongly for a restriction of trading hours. The industry actors argued unanimously and strongly against this measure, using various lines of arguments that have clear similarities with those on scientific research evidence. First, in their response to the police and others, who argued that restricted trading hours had led to fewer police reported assaults, several industry actors questioned the validity of police statistics on violence, for instance claiming that the violence statistics presented by the police do not reflect the real trends in violence, and also that a number of other factors than trading hours have impacted on the local violence statistics..

*“Is it really a true decrease in violence in [city], and what is the true cause of the decrease? This can only be discussed when we have access to – and can perform independent analyses of – the real data. However, the police refuses to publish these data.”* (Nine individual hospitality industry representatives, in a common opinion piece to a local newspaper, January, 2010).

Several actors, both among the individual bar owners and among the hospitality industry trade organizations, claimed that the police exaggerated the violence problem.

*“There is not as much trouble around bars and pubs as the police claim.” (SH, former night club owner and conservative local politician, January 16, 2012).*

Some also accused the police of manipulating their data or their presentation of the police statistics. The similarities with tactics used in critiques of the research at the national level, thus extended also to attacking the integrity of those providing the evidence.

*“The police only uses the data that support their own views, which leads to a biased presentation.[ ] I suspect that the police both over-report and under-report in order to obtain a desired basis for their recommendations to the policy makers.” (MM, Bar manager, June 26, 2011).*

Cherry picking of evidence was also seen among industry actors at the local level. Some referred to experiences in other cities or regions, suggesting that trading hours had no impact on violence, or even the opposite effect, thereby selecting anecdotal evidence to support their views.

Others refuted any impact of restricted trading hours by dismissing alcohol serving as the source of the problem, and they claimed that violence and nuisance were often attributable to drugs rather than alcohol, or that drinking prior to going to bars was the cause of late night violence in the city centres rather than on-premise drinking.

*“Many of those who cause trouble, have something quite different than alcohol in their blood, and we do not have powder in our taps.” (AK, bar owner, July 18, 2012).*

*“Are we [who work] in the night time economy business responsible for violence, given that only a small fraction of the alcohol is sold by us? [ ] We experience that people drink a lot before going to the city centre.” (HCS, pub owner, April 24, 2012).*

Overall, the industry trade organizations were less prominent in debates at the local level as compared to the debate on national restrictions. At the local level, individual bar and pub owners and their local associations were more prominent. The trade organizations’ arguments at the local level reflected those used in the national debate, although in this context, they emphasized to a larger extent the importance of the local authorities’ capacity to assess and decide on local matters. The individual bar and pub owners responded more often to local police statistics than to research on trading hours and violence and in doing so, they sometimes cherry picked examples to illustrate their points of view.

The trade organizations had sufficient resources to prepare, nuance and substantiate their arguments on evidence and also to convey them through more numerous and more significant channels than local actors. Other actors did also use the phrases and arguments already produced by the trade organization actors. But, rather than providing a mere echo, they often presented their arguments along with their own personal testimonies of knowledge of the business and the problem at hand, which may have strengthened credibility. In the two cities where industry actors claimed lack of evidence on closing hours and violence in their hearing statements, the political decisions differed; in one city the proposed restriction was not adopted, and in the other city, restricted closing time was continued.

## Discussion

Industry actors employed various strategies to shape perceptions of evidence by conveying their preferred interpretations regarding on-premise trading hours and violence. The relevance of the international research literature to the impacts of smaller changes to closing times was questioned first at the national level. After the publication of evidence demonstrating impacts of such changes in Norway, industry actors attacked the study and systematically cast doubt on the evidence, employing a range of strategies which are used by industry actors in other areas [11] (see Table 2 for a summary). At the local level, industry actors criticized both research evidence and police statistics, but they were less versatile in their ways of criticizing evidence, and police statistics were much more important than research evidence in the local debates. Both in the hearings statements in policy making processes and in media articles, we find that the larger organizations typically were better resourced and more sophisticated in their shaping of the evidence, as compared to the individual actors and local organizations. Alcohol industry actors repeated the same or similar arguments recurrently in different media interviews and newspaper contributions, even using identical phrases. Many of the key phrases and arguments originated in the two reports commissioned by the trade organizations. On several occasions, industry representatives published the same opinion piece in several different national and regional newspapers, or press releases were simply reproduced without editing.

In line with the existing research literature on industry tactics to shape perceptions of available evidence (see [33] for tobacco industry) we also found commissioning of attacks critiquing the research, emphasizing disagreements among experts, in this case constructing the status of expert to mean informed opinion, and not referring to scientific debates, focusing on doubt, complexity, and confounding; and cherry picking of data. In addition to attacking the evidence directly, we found attacks on the researchers and police integrity, a strategy which has also been used by for instance the food industry and the tobacco industry, when claiming that researchers had a political agenda [34, 35].

There has been limited prior study of how alcohol industry actors shape evidence potentially harmful to the industry in the processes of policy making [16, 17]. Alcohol industry actors routinely claim to be committed to evidence informed policy, yet consistently misrepresent evidence, apparently because they oppose the market regulatory approaches which evidence shows are most likely to be effective [15]. Doubt about evidence interferes with evidence use by policy makers, and this is a key feature of the existing research in this area. Studies suggest that the extent to which scientific evidence has been used in policy making may be pivotal in explaining the divergence in national alcohol policy between England and Scotland for example [15, 36]. The importance of the framing of alcohol policy evidence in line with policy preferences has been examined in existing studies, particularly in respect of the political strategies of the global producers [16, 37]. Here we find national level trade organisations using similar approaches to framing research evidence, after initially discounting the relevance of the international literature.

Study limitations are several. First, alcohol industry actors have clearly responded through a broader range of channels than those covered by our data set, including TV debates and interviews, and directly lobbying politicians and other policy actors. Second, many industry actors may have chosen, as a deliberate strategy, to ignore research/police statistics and this kind of strategy could not be revealed in our data set, which concerns what was done rather than what was not done. Thus, the full range of strategies in the handling of evidence employed by industry actors likely exceed those identified in the present study. Finally, the question as to whether, or to what extent, the shaping of evidence through industry activities made an impact in the policy making processes at the national and local levels, lies beyond the scope of the present study. The use of research based knowledge and other evidence of the effects of policy measures is far from simple and solely instrumental in policy making processes, it is also the subject of, and reflects the balance of forces in, political contestation [38–41].

The present study contributes to the existing evidence base specifically on alcohol industry actors by drawing attention to the ways in which strategic approaches to evidence management vary flexibly over time as the evidence itself changes, and as do the policy circumstances. The circumstances of this study are of a policy controversy operating contemporaneously at both national and local levels where scientific evidence published by the first author was a high profile feature of the debate. Other policy debates may involve scientific and other forms of evidence in less prominent ways. The handling of evidence studied here is one component of higher level political strategies which frame the issues with which policy making contends in ways favourable to industry interests so as to defeat unfavoured proposals, such as this one [16]. This framing activity was here led by trade organisations as there are no dedicated social aspects organizations in Norway, as there are in other countries [42]. In other countries this political function is performed by social aspects organizations where they exist [37]. There are also major differences in cultures of evidence use at the local and national level [43], with local data sources and actor types more prominent in the former, a high level of organisation and co-ordination in the latter, and some evidence of interplay between the two in this instance.

As part of the research process, researchers get involved in research dissemination, knowledge exchange and various aspects of policy making. This study demonstrates that, notwithstanding reflexivity considerations, there is important value in not only observing and promoting the use of research evidence in policy making, but in also studying it. Alcohol policy researchers are well placed to identify misuses of evidence in the strategic management of research by industry actors and other vested interests. They are thus well placed to assist policy actors. Alcohol industry actors rhetorically distance themselves from tobacco industry actors in their efforts to influence policy, which have otherwise shared obvious similarities [16, 44], notwithstanding cross-ownership [45]. National governments and other public health policy actors will benefit from paying close attention to the similarities between these two categories [46, 47], and researchers can contribute by furthering understanding of the similarities and differences. Further study of industry actor policy involvement, specifically including use of evidence therein, is an important aspect to developing public health countermeasures to alcohol industry influence on policy [48].

## Conclusion

Alcohol industry actors employed a range of strategies to shape the use of the various types of evidence on on-premise trading hours and violence to advance their own interests. The particular strategies and arguments changed over time as new data and research became available, and also varied between the national and the local levels and by categories of industry actors. There is a need to better understand how the handling of different forms of evidence is accommodated within the political strategies of a range of industry actors in respect of particular policy issues and forms of evidence, at different levels of policy making and in varying policy contexts.

## Abbreviation

RN study: Rossow & Norström, 2012 study. Reference no 25
